# Analysis of white matter tract integrity using diffusion kurtosis imaging reveals the correlation of white matter microstructural abnormalities with cognitive impairment in type 2 diabetes mellitus

**DOI:** 10.3389/fendo.2024.1327339

**Published:** 2024-02-29

**Authors:** Jie Gao, Peichun Pan, Jing Li, Min Tang, Xuejiao Yan, Xin Zhang, Man Wang, Kai Ai, Xiaoyan Lei, Xiaoling Zhang, Dongsheng Zhang

**Affiliations:** ^1^ Department of MRI, Shaanxi Provincial People’s Hospital, Xi’an, China; ^2^ Department of Graduate, Shaanxi University of Chinese Medicine, Xianyang, China; ^3^ Department of Graduate, Xi’an Medical University, Xi’an, China; ^4^ Department of Clinical Science, Philips Healthcare, Xi’an, China

**Keywords:** type 2 diabetes mellitus, diffusion kurtosis imaging, microstructure, white matter tract integrity, neuroimaging

## Abstract

**Background:**

This study aimed to identify disruptions in white matter integrity in type 2 diabetes mellitus (T2DM) patients by utilizing the white matter tract integrity (WMTI) model, which describes compartment-specific diffusivities in the intra- and extra-axonal spaces, and to investigate the relationship between WMTI metrics and clinical and cognitive measurements.

**Methods:**

A total of 73 patients with T2DM and 57 healthy controls (HCs) matched for age, sex, and education level were enrolled and underwent diffusional kurtosis imaging and cognitive assessments. Tract-based spatial statistics (TBSS) and atlas-based region of interest (ROI) analysis were performed to compare group differences in diffusional metrics, including fractional anisotropy (FA), mean diffusivity (MD), axonal water fraction (AWF), intra-axonal diffusivity (D_axon_), axial extra-axonal space diffusivity (D_e,//_), and radial extra-axonal space diffusivity (D_e,⊥_) in multiple white matter (WM) regions. Relationships between diffusional metrics and clinical and cognitive functions were characterized.

**Results:**

In the TBSS analysis, the T2DM group exhibited decreased FA and AWF and increased MD, D_e,∥_, and D_e,⊥_ in widespread WM regions in comparison with the HC group, which involved 56.28%, 32.07%, 73.77%, 50.47%, and 75.96% of the mean WM skeleton, respectively (*P* < 0.05, TFCE-corrected). D_e,⊥_ detected most of the WM changes, which were mainly located in the corpus callosum, internal capsule, external capsule, corona radiata, posterior thalamic radiations, sagittal stratum, cingulum (cingulate gyrus), fornix (stria terminalis), superior longitudinal fasciculus, and uniform fasciculus. Additionally, D_e,⊥_ in the genu of the corpus callosum was significantly correlated with worse performance in TMT-A (β = 0.433, *P* < 0.001) and a longer disease duration (β = 0.438, *P* < 0.001).

**Conclusions:**

WMTI is more sensitive than diffusion tensor imaging in detecting T2DM-related WM microstructure abnormalities and can provide novel insights into the possible pathological changes underlying WM degeneration in T2DM. D_e,⊥_ could be a potential imaging marker in monitoring disease progression in the brain and early intervention treatment for the cognitive impairment in T2DM.

## 1 Introduction

Type 2 diabetes (T2DM) is the most common metabolic disease with a high incidence worldwide. According to the latest data from the International Diabetes Federation in 2019, the number of people with T2DM worldwide was 463 million, and the global prevalence rate of T2DM was 9.3% (1). Brain damage caused by persistent hyperglycemia, also defined as diabetic encephalopathy, which includes changes in neurophysiology and brain structures as well as the resultant cognitive impairment involving memory, attention and executive functions (2, 3), has recently attracted increasing attention. Cognitive impairment in T2DM is usually not necessarily accompanied by subjective cognitive complaints (4). Furthermore, with the increasing prevalence of diabetes and an aging population, the incidence of cognitive impairment is expected to increase gradually, posing great challenges for future health effects. However, the neuropathological basis of the cognitive impairment associated with T2DM is still unclear.

A series of recent neuroimaging studies have revealed that T2DM is accompanied by cerebral microstructural impairments related to cognitive decline. In addition to gray matter atrophy, white matter (WM) microstructural abnormalities are also believed to play a prominent role in T2DM-induced cognitive impairment (5–8). Diffusion tensor imaging (DTI) can provide an effective and quantitative method to delineate WM microstructural changes. The integrity of the WM may be inferred on the basis of changes in DTI-derived parameters, including fractional anisotropy (FA), mean diffusivity (MD), axial diffusivity (AD), and radial diffusivity (RD), potentially providing evidence to evaluate pathological changes in T2DM. The existing studies based on DTI have confirmed extensive WM microstructural abnormalities in T2DM, and some important regions of the WM have also been demonstrated to be correlated with neuropsychological performance (9–11). Furthermore, the WM changes in T2DM mainly manifested as decreased FA and increased MD and RD, which may reflect axonal degeneration, myelin breakdown, or other factors. However, due to the non-specificity of DTI measures, this approach showed limitations in determining the altered structures underlying these diffusional abnormalities. In addition, since the DTI model assumes a Gaussian distribution of water-diffusion processes with a mono-exponential signal decay, it cannot easily characterize crossing fibers, leading to limited information.

Diffusional kurtosis imaging (DKI) is a clinically feasible extension of DTI that examines the additional contribution of non-Gaussian diffusion effects to provide additional information on brain microstructural complexity (12). The derived metrics from DKI, including mean kurtosis (MK), axial kurtosis (K_∥_), and radial kurtosis (K_⊥_), can measure the complexities of structures and can be used to assess white matter and gray matter simultaneously. Although effective, DKI metrics can only probe voxel-level diffusion that mingles the effects of heterogeneous microenvironments but are not specific to features of microstructure, since they are calculated based on a model of brain tissue microstructure involving a single compartment. This limitation makes it difficult to interpret potential neuropathological changes. For example, the reduced MK, K_⊥_ and K_∥_ could be caused by a reduction in axons, demyelination and an increase of free water in the outer space of the axon (or both). Subsequently, a two-compartment non-exchange diffusion model based on DKI has been proposed to identify compartment-specific white matter tract integrity (WMTI) in the intra-axonal spaces and extra-axonal spaces (13). Notably, the derived metrics, including the axonal water fraction (AWF), intra-axonal diffusivity (D_axon_), and extra-axonal axial and radial diffusivities (D_e,//_and D_e,⊥_), offer enhanced insights into the microstructural features. D_axon_ serves as a biomarker with high sensitivity and specificity for detecting axonal abnormalities. D_e,//_and D_e,⊥_indicates a relatively free water content in the extra-axonal space, as well as the integrity of myelin sheath. AWF quantifies the proportion of water within the axons relative to the total water fraction encompassing both intra- and extra-axonal water. These metrics greatly improve the characterization of subtle tissue microstructural changes, and have been demonstrated to deepen the understanding of WM alterations in various neurological disorders, such as Alzheimer’s disease (14), multiple sclerosis (15), aging (16), and mild traumatic brain injury (17).Since WMTI metrics can reveal more specific diffusivity changes from water inside the axons (potentially glial processes) and outside axons (excluding water from myelin sheath and interstitial space), this study aims to (1) determine whether WMTI metrics are more sensitive than DTI metrics in investigating WM microstructural changes in patients with T2DM; (2) further speculate the possible neuropathological changes associated with cognitive impairment in T2DM patients based on additional information provided by WMTI metrics; and (3) investigate the correlations between altered WMTI metrics and clinical/cognitive variables to identify the imaging biomarkers of cognitive decline in patients with T2DM.

## 2 Materials and methods

### 2.1 Participants

The study was conducted in accordance with the Helsinki declaration. The Human Ethics Committee of Shaanxi Provincial Peoples Hospital approved all experimental procedures after receiving informed consent (the ethical committee protocol number: 2022K101).

Seventy-three patients with T2DM were recruited from the Department of Endocrinology of Shaanxi Provincial People’s Hospital, and 57 healthy controls (HCs) matched for age, sex, and education level were obtained from our health examination center. All the participants were between 40 and 70 years of age, right-handed, and educated for at least six years. The T2DM patients met the standard criteria proposed by the American Diabetes Association (18) and without a history of hypoglycemia or hyperglycemia. The other inclusion criteria in the HC group were as follows: (1) no symptoms or a family history of diabetes; (2) fasting glucose < 7.0 mmol/L; and (3) glycosylated hemoglobin A1c (HbA1c) level < 6.0%. The exclusion criteria for both groups were as follows: (1) history of neurological disorders, such as cerebral infarctions, brain tumors, vascular malformations, or traumatic brain injury; (2) illicit substance abuse, alcohol abuse, or psychiatric disorders; (3) any other systemic disease irrelevant to diabetes and affecting cognition; (4) periventricular or deep WM hyperintensities with a Fazekas score > 1 on T2-weighted fluid-attenuated inversion recovery (FLAIR) images; and (5) inability to complete MRI examinations or unsatisfactory MRI images.

### 2.2 Clinical data and neuropsychological test results

All participants’ clinical data, including information regarding their age, sex, educational level, blood pressure, height, weight, and body mass index (BMI), were obtained from medical records and questionnaires. Laboratory tests, including blood biochemical analysis and evaluation of fasting plasma glucose (FPG) and HbA1c levels were performed and their results were recorded. All participants underwent the following series of neuropsychological assessments: Mini-Mental State Examination (MMSE) and Montreal Cognitive Assessment (MoCA) were used to assess general cognitive function; Trail-Making Test A (TMT-A) reflected cognitive processing skills (psychomotor speed, processing speed, and visuospatial skills) and attention; Trail-Making Test B (TMT-B) provided information regarding executive function; Auditory Verbal Learning Test (AVLT) was used to evaluate episodic memory; and Clock Drawing Test (CDT) was performed to evaluate visuospatial skills. Neuropsychological tests were performed by a psychiatrist trained in systematic testing.

### 2.3 Image acquisition

Conventional MRI and DKI data were obtained using a 3.0 T MR scanner (Ingenia, Philips Medical Systems, the Netherlands) equipped with a 16-channel phase-array head coil. Conventional MRI, including sagittal three-dimensional T1-weighted imaging (repetition time [TR]/excitation time [TE] = 7.5/3.5 ms, field of view [FOV] = 250 × 250 mm^2^, matrix = 256 × 256, slice thickness = 1 mm) and T2 FLAIR (TR/TE = 6000/150 ms, FOV = 230 × 230 mm^2^, matrix = 320 × 320, slice thickness = 6 mm) were used to identify visible brain lesions. DKI was performed using a spin-echo echo-planar imaging sequence with the following parameters: TR/TE = 6000/150 ms, slice thickness = 6 mm, FOV = 224 × 224 mm^2^, matrix = 112 × 112, number of excitations = 1, b value = 0, 1000, 2000 s/mm^2^, 32 diffusion encoding directions for each nonzero b value with the same distribution. The gradient directions were uniformly distributed by performing the electro-static repulsion method (19, 20).

### 2.4 Data processing

Data pre-processing included the following steps. First, the image format was converted from DICOM to NIFTI. Second, non-brain tissues, such as the scalp and skull, were removed using the Brain Extraction Tool (BET), which was performed in FMRIB’s Software Library (FSL). Third, the diffusion-weighted images were realigned to the non-weighted b0 images to correct head motion and eddy current-induced distortions (also a tool of FSL). Finally, artifact-corrupted diffusion-weighted imaging scans were excluded by using an automated method (21). Only two b values (0 and 1000 s/mm^2^) were used for DTI fitting, which was performed with the FMRIB diffusion toolbox (FDT), and four DTI parameters, including FA and MD, were obtained. The kurtosis tensor was estimated by using a constrained weighted linear least squares method (CWLLS) (22, 23) in MATLAB (MathWorks, Natick, Massachusetts). Then, the WMTI metrics, including AWF, D_axon_, D_e,∥_, and D_e,⊥_, were estimated using the algorithms of the WMTI model (13) with an in-house program implemented in MATLAB.

### 2.5 TBSS analysis

FSL with tract-based spatial statistics (TBSS, part of FSL) was used to analyze all of the above DTI and WMTI metrics and compare intergroup differences in WM. All participants’ FA images were first aligned to a common target FMRIB58_FA in the MNI 152 standard space using nonlinear registration. Then, the mean FA image and its skeleton were generated. The aligned FA image of each participant was projected onto the mean FA skeleton (threshold = 0.2). The resulting FA skeleton images were fed into voxel-wise cross-subject statistics to identify intergroup differences in major WM tracts. The number of permutations was set at 5000. The results were corrected for multiple comparisons by controlling the family-wise error rate after threshold-free cluster enhancement (TFCE). TBSS analysis was also performed for other DTI and WMTI metrics parameters. The results of all tests were considered to be significant at *P* < 0.05. The Johns Hopkins University (JHU) WM ICBM-DTI-81 WM labels atlas in FSL (http://fsl.fmrib.ox.ac.uk/fsl/fslwiki/Atlases) was used to label significant tracts.

### 2.6 ROI analysis

ROI analysis was also performed based on the Johns Hopkins University WM label atlas, in which the entire WM was parceled into 48 ROIs. The FA, MD, and WMTI metrics were measured in these ROIs and reported as mean ± standard deviation. The ROI-based comparisons between the T2DM and the HC groups were further performed in regions that showed significant differences in the TBSS analysis described above.

The correlations between DTI and WMTI metrics within the resultant ROIs and the neuropsychological assessment scores, disease duration, and HbA1c level in T2DM patients were analyzed by using the multiple linear regression analyses, with age, sex, and years of education as the covariates. The Bonferroni correction was applied to correct for multiple comparisons. The regression coefficients (β) and *P* values were calculated.

### 2.7 Statistical analysis

The demographic, clinical, and neuropsychological data were analyzed by using SPSS (*SPSS version 20.0*, IBM, USA). The measurement data were reported as mean ± standard deviation, and categorical data were presented as frequencies and percentages. Intergroup comparisons of these data were performed using Student’s *t* tests or χ^2^ tests as appropriate. All tests were taken to be significant at *P* < 0.05.

## 3 Results

### 3.1 Clinical and neuropsychological data

Clinical and neuropsychological data are presented in **Table 1**. The T2DM and control groups showed no significant differences in sex, age, years of education, BMI, blood pressure, and the total cholesterol, triglyceride, and low-density lipoprotein levels. The T2DM group showed higher HbA1c and FPG levels and lower high-density lipoprotein levels than the HC group (*P* < 0.001). Of the 73 patients with T2DM, 32 were receiving insulin treatment, and the remaining 41 patients were receiving oral hypoglycemic agents. The T2DM group showed poorer performance in general cognitive function (MMSE, *P* = 0.016; MoCA, *P* < 0.001). In addition, the T2DM group showed significantly worse results in the cognitive domains of attention, executive function, and episodic memory than the HCs.

**Table 1:** Clinical characteristics of participants.

Clinic information	T2DM (n = 73)	HC (n =57)	T/χ^2^ value	*P* value
Gender (male/female)	55/18	38/19	1.183	0.277^#^
Age (years)	55.64 ± 7.65	54.18 ± 5.64	-1.260	0.210
Formal education (years)	13.77 ± 2.40	14.05 ± 2.52	-0.914	0.361
BMI (kg/m^2^)	25.16 ± 2.73	24.46 ± 2.72	-1.462	0.146
Systolic pressure (mmHg)	127.36 ± 15.92	125.70 ± 13.41	-0.342	0.732
Diastolic pressure (mmHg)	79.08 ± 11.14	82.09 ± 10.73	1.551	0.123
Total cholesterol (mmol/L)	4.44 ± 1.15	4.75 ± 0.77	1.799	0.074
Triglycerides (mmol/L)	2.05 ± 1.35	1.73 ± 1.07	-1.432	0.154
HDL (mmol/L)	1.04 ± 0.27	1.31 ± 0.29	5.572	<0.001
LDL (mmol/L)	2.61 ± 0.81	2.82 ± 0.67	1.595	0.113
Fasting glucose (mmol/L)	7.81 ± 2.28	5.07 ± 0.56	-9.931	<0.001
HbA1c (%)	7.85 ± 2.05	5.44 ± 0.43	-9.011	<0.001
Diabetes duration (year)	8.34 ± 5.93	/	/	/
Insulin use(n/%)	32/43.84	/	/	/
MMSE	28.09 ± 1.64	28.82 ± 1.14	-2.407	0.016
MoCA	24.81 ± 2.64	27.59 ± 0.76	-6.624	<0.001
TMT-A(s)	82.59 ± 25.27	73.09 ± 25.14	-2.132	0.035
TMT-B(s)	163.15 ± 49.02	137.19 ± 56.79	-2.794	0.006
CDT	25.77 ± 5.92	26.04 ± 3.12	-1.699	0.089
AVLT-total	40.44 ± 6.67	43.09 ± 5.65	2.401	0.018
AVLT-delay recall	8.19 ± 2.82	8.51 ± 2.32	0.686	0.494

Data are presented as mean ± standard deviation or number (%) unless otherwise indicated. BMI, body mass index; HDL, high density lipoprotein; LDL, low density lipoprotein; HbA1c, glycated hemoglobin; MMSE, Mini-Mental State Examination; MoCA: Montreal Cognitive Assessment; TMT-A, Trail Making Test A; TMT-B, Trail Making Test B; CDT, clock drawing test; AVLT, Auditory Verbal Learning Test. ^#^The P value was obtained using a χ^2^ test. / means there is no data in the corresponding cells.

### 3.2 WM skeleton voxel-wise TBSS comparisons

**Figure 1:**
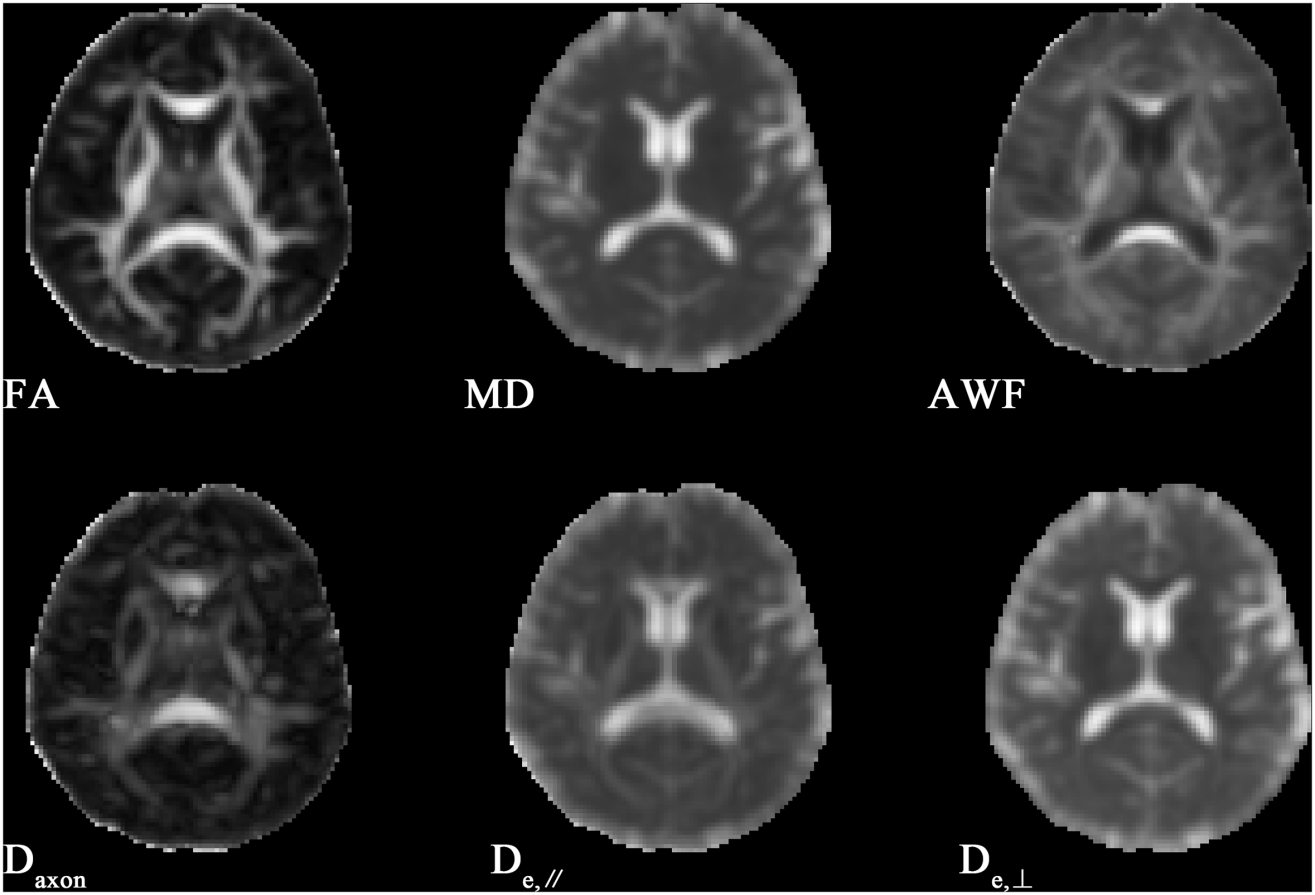
Illustrations of FA, MD and WMTI metrics in slice of basal ganglia of a healthy control.

**Figure 2:**
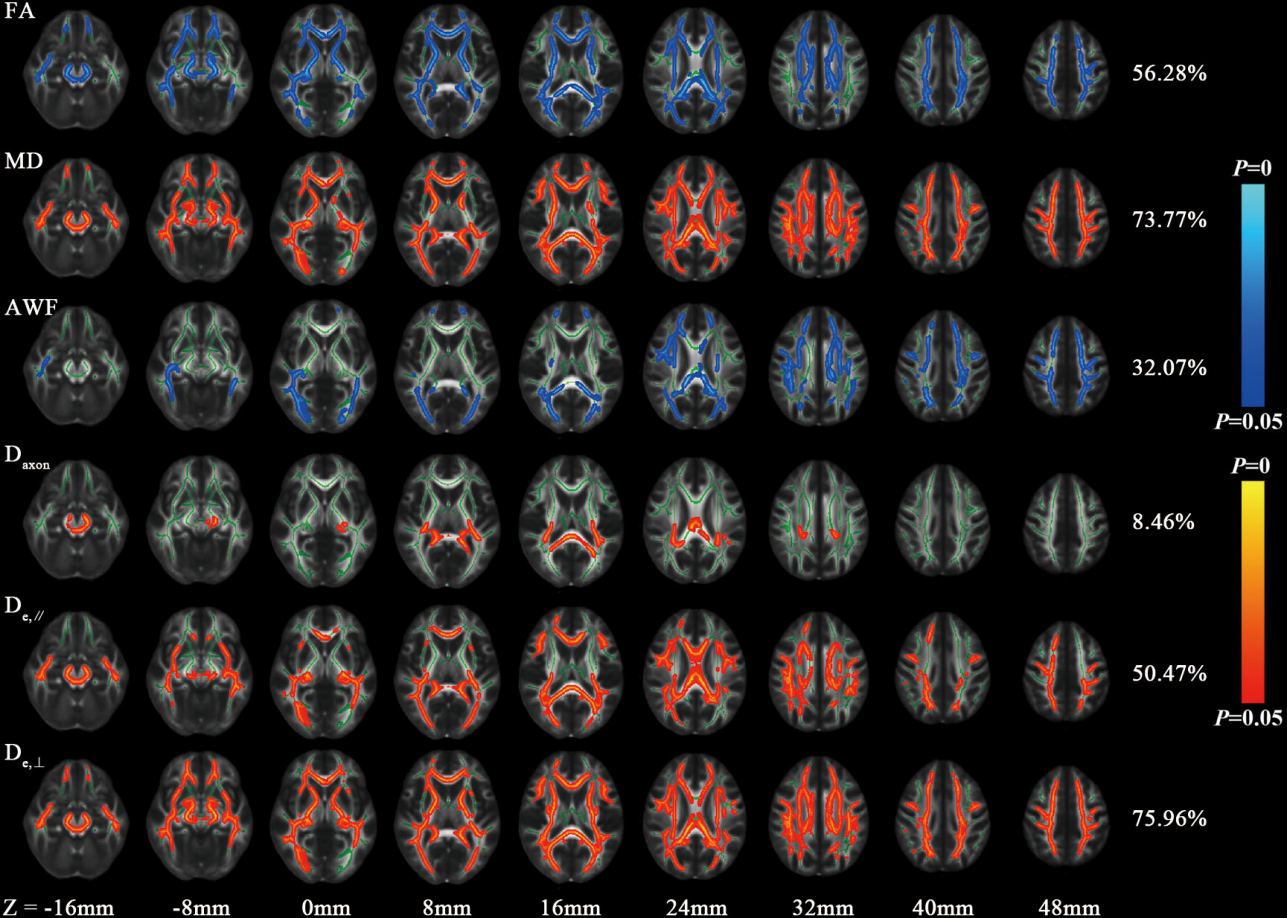
Differences of FA, MD and WMTI metrics between HC and T2DM group by voxel-wise TBSS analysis. Regions colored light blue-blue represent significantly decreased voxels (*P*< 0.05) in the T2DM group compared with the HC group, while regions colored yellow-red represent significantly increased voxels (*P*< 0.05). These have been overlaid on the mean FA template with a mean skeleton (regions colored green). Numbers on the right of each row are percentage of voxels that changed in the whole brain.

**Figure 1** shows the derived FA, MD, AWF, D_axon_, D_e,∥_ and D_e,⊥_ maps from an HC for illustration. In TBSS analysis, the T2DM group exhibited significantly deceased FA and AWF and increased MD, D_e,∥_, and D_e,⊥_ over widespread WM regions (as shown in **Figure 2**), which involved 56.28% (33874/60190 voxels), 32.07% (19303/60190 voxels), 73.77% (44404/60190 voxels), 50.47% (30380/60190 voxels), and 75.96% (45721/60190 voxels) of the mean WM skeleton, respectively (*P* < 0.05, TFCE-corrected). Additionally, increased D_axon_ values were only found in some discrete WM regions in the T2DM group compared with HC group. To recognize the more robust and severely impaired WM tracts in T2DM patients, additional investigations with more strict criteria (*P* < 0.01, TFCE-corrected) were performed in the TBSS analysis. These results also showed that D_e,⊥_ exhibited more involved WM regions (43.83%) than FA and MD (39.66% and 29.45%, respectively), which were mainly located in the whole corpus callosum (CC), internal capsule (IC), external capsule (EC), corona radiata (CR), posterior thalamic radiations (PTR), sagittal stratum (SS), cingulum (cingulate gyrus), fornix (stria terminalis), superior longitudinal fasciculus (SLF), and uncinate fasciculus (UF).

### 3.3 Group differences in the atlas-based tract ROIs

**Figure 3:**
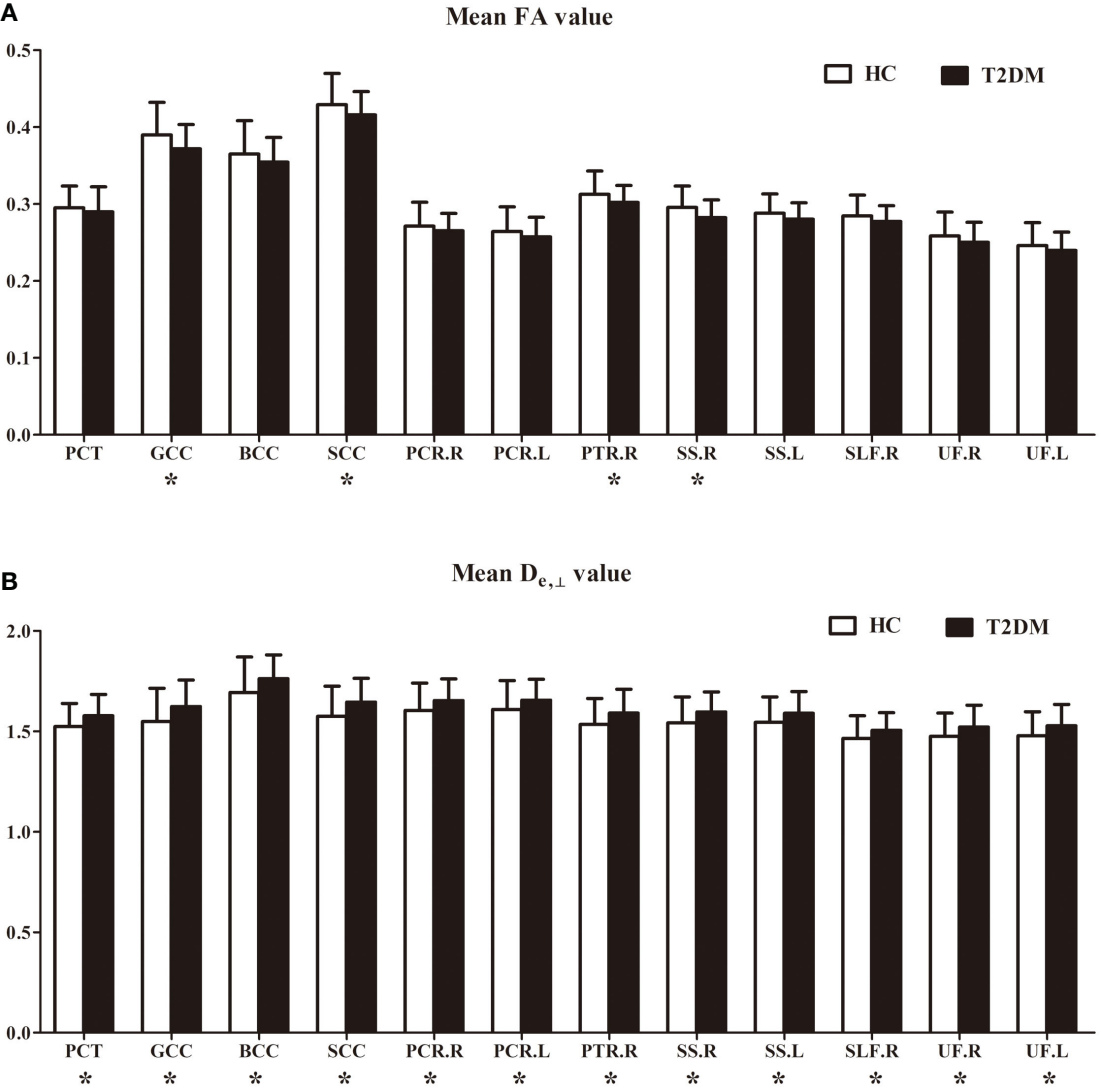
FA **(A)** and D_e, ⊥_ **(B)** differences in some white matter fiber tracts. PCT, pontine crossing tract; GCC, genu of corpus callosum; BCC, body of corpus callosum; SCC, splenium of corpus callosum; PCR, posterior corona radiata; PTR, posterior thalamic radiation; SS, sagittal stratum; SLF, superior longitudinal fasciculus; UF, uncinate fasciculus; R, right; L, left. *indicates a significant difference between the two groups at a significance level of 0.05.

In the ROI-based quantitative analysis, the T2DM patients again showed similar changes in FA, AWF, MD, D_axon_, D_e,∥_, and D_e,⊥_ in most WM regions as in the TBSS analysis. In the 48 WM tracts defined in the JHU WM ICBM-DTI-81 WM labels, more regions had increased D_e,⊥_ (28/48 regions) than decreased FA (18/48 regions) in the T2DM group (shown in **Tables 2**, **3**). **Figure 3** summarizes the FA and D_e,⊥_ differences in some WM fiber tracts. Notably, only D_e,⊥_ differences were found in some crossing fibers, including the pontine crossing tract, bilateral PTR, right SLF, and bilateral UF.

**Table 2:** White matter integrity differences of FA and D_e,⊥_ in disrupted WM tracts between T2DM and HC group during ROI-based statistical analysis.

Fiber tract	FA	D_e,⊥_
PCT	–	YES
GCC	YES	YES
BCC	–	YES
SCC	YES	YES
CST	Bilateral	Bilateral
ML	Bilateral	Bilateral
CP	Bilateral	Bilateral
ALIC	R	R
PLIC	R	R
ACR	R	R
SCR	Bilateral	Bilateral
PCR	–	Bilateral
PTR	R	R
Sagittal stratum	R	Bilateral
EC	R	R
Cingulum (cingulate gyrus)	Bilateral	Bilateral
FORNIX	–	Bilateral
SLF	–	R
UF	–	Bilateral
Number of ROIs	18	28

YES means diffusional metrics of this ROI showed significant difference between T2DM and HC groups, since it is a whole region which can’t be divided into left and right sides. - means these is no difference between the two groups. R means the right side of ROI region, and bilateral means both the right and left sides. PCT, pontine crossing tract; GCC, genu of corpus callosum; BCC, body of corpus callosum; SCC, splenium of corpus callosum; CST, corticospinal tract; ML, medial lemniscus; CP, cerebral peduncle; ALIC, anterior limb of internal capsule; PLIC, posterior limb of internal capsule; ACR, anterior corona radiata; SCR, superior corona radiata; PCR, posterior corona radiata; PTR, posterior thalamic radiation; EC, external capsule; SLF, superior longitudinal fasciculus; UF, uncinate fasciculus.

**Table 3:** Differences of FA and D_e,⊥_ (means ± SDs) between T2DM and HC group during ROI-based statistical analysisROI.

	FA				D_e,⊥_(×10^-3^mm/s^2^)			
	HC	T2DM	t	p	HC	T2DM	t	p
PCT	0.30 ± 0.03	0.29 ± 0.03	0.964	0.337	1.52 ± 0.12	1.58 ± 0.11	-2.780	0.006
GCC	0.39 ± 0.04	0.37 ± 0.03	2.675	0.009	1.55 ± 0.17	1.62 ± 0.13	-2.846	0.005
BCC	0.37 ± 0.04	0.35 ± 0.03	1.551	0.124	1.69 ± 0.18	1.76 ± 0.12	-2.546	0.013
SCC	0.43 ± 0.04	0.42 ± 0.03	2.133	0.035	1.58 ± 0.15	1.65 ± 0.12	-2.995	0.003
CST.R	0.31 ± 0.04	0.29 ± 0.03	3.495	0.001	1.67 ± 0.17	1.77 ± 0.16	-3.584	<0.001
CST.L	0.33 ± 0.03	0.31 ± 0.03	3.394	0.001	1.61 ± 0.13	1.69 ± 0.14	-3.588	<0.001
ML.R	0.37 ± 0.03	0.35 ± 0.03	3.688	<0.001	1.53 ± 0.12	1.61 ± 0.12	-3.722	<0.001
ML.L	0.34 ± 0.03	0.33 ± 0.02	3.784	<0.001	1.57 ± 0.13	1.65 ± 0.12	-3.690	<0.001
CP.R	0.38 ± 0.03	0.36 ± 0.03	3.547	0.001	1.49 ± 0.13	1.57 ± 0.11	-3.619	<0.001
CP.L	0.39 ± 0.03	0.37 ± 0.02	3.194	0.002	1.50 ± 0.12	1.57 ± 0.10	-3.936	<0.001
ALIC.R	0.32 ± 0.03	0.31 ± 0.03	3.183	0.002	1.35 ± 0.13	1.41 ± 0.10	-2.894	0.004
PLIC.R	0.42 ± 0.04	0.40 ± 0.03	3.057	0.003	1.29 ± 0.12	1.33 ± 0.09	-2.374	0.019
ACR.R	0.26 ± 0.03	0.24 ± 0.02	4.209	<0.001	1.44 ± 0.13	1.49 ± 0.10	-2.667	0.009
SCR.R	0.29 ± 0.03	0.28 ± 0.02	3.404	0.001	1.42 ± 0.12	1.47 ± 0.09	-2.654	0.009
SCR.L	0.30 ± 0.03	0.29 ± 0.02	2.837	0.005	1.43 ± 0.12	1.47 ± 0.09	-2.256	0.026
PCR.R	0.27 ± 0.03	0.27 ± 0.02	1.211	0.229	1.60 ± 0.14	1.65 ± 0.11	-2.304	0.023
PCR.L	0.26 ± 0.03	0.26 ± 0.03	1.390	0.167	1.61 ± 0.14	1.65 ± 0.10	-2.088	0.039
PTR.R	0.31 ± 0.03	0.30 ± 0.02	2.311	0.022	1.54 ± 0.13	1.59 ± 0.12	-2.573	0.011
SS.R	0.30 ± 0.03	0.28 ± 0.02	2.973	0.004	1.54 ± 0.13	1.60 ± 0.10	-2.731	0.007
SS.L	0.29 ± 0.03	0.28 ± 0.02	1.910	0.058	1.55 ± 0.13	1.59 ± 0.11	-2.204	0.029
EC.R	0.26 ± 0.03	0.25 ± 0.02	2.048	0.043	1.41 ± 0.12	1.46 ± 0.10	-2.394	0.018
Cingulum.R	0.26 ± 0.02	0.25 ± 0.02	2.262	0.025	1.42 ± 0.11	1.48 ± 0.08	-3.083	0.003
Cingulum.L	0.27 ± 0.03	0.26 ± 0.02	2.581	0.011	1.43 ± 0.12	1.48 ± 0.08	-3.011	0.003
FORNIX.R	0.28 ± 0.03	0.27 ± 0.02	1.727	0.087	1.55 ± 0.14	1.63 ± 0.12	-3.375	0.001
FORNIX.L	0.29 ± 0.03	0.28 ± 0.02	1.242	0.217	1.58 ± 0.14	1.66 ± 0.12	-3.458	0.001
SLF.R	0.28 ± 0.03	0.28 ± 0.02	1.673	0.097	1.46 ± 0.11	1.51 ± 0.09	-2.299	0.023
UF.R	0.26 ± 0.03	0.25 ± 0.03	1.619	0.108	1.48 ± 0.12	1.52 ± 0.11	-2.309	0.023
UF.L	0.25 ± 0.03	0.24 ± 0.02	1.367	0.174	1.48 ± 0.12	1.53 ± 0.11	-2.509	0.013

PCT, pontine crossing tract; GCC, genu of corpus callosum; BCC, body of corpus callosum; SCC, splenium of corpus callosum; CST, corticospinal tract; ML, medial lemniscus; CP, cerebral peduncle; ALIC, anterior limb of internal capsule; PLIC, posterior limb of internal capsule; ACR, anterior corona radiata; SCR, superior corona radiata; PCR, posterior corona radiata; PTR, posterior thalamic radiation; SS, sagittal stratum; EC, external capsule; SLF, superior longitudinal fasciculus; UF, uncinate fasciculus; R, right; L, left.

### 3.4 Correlations between ROI-wise diffusion metrics and cognitive and clinical data

**Figure 4:**
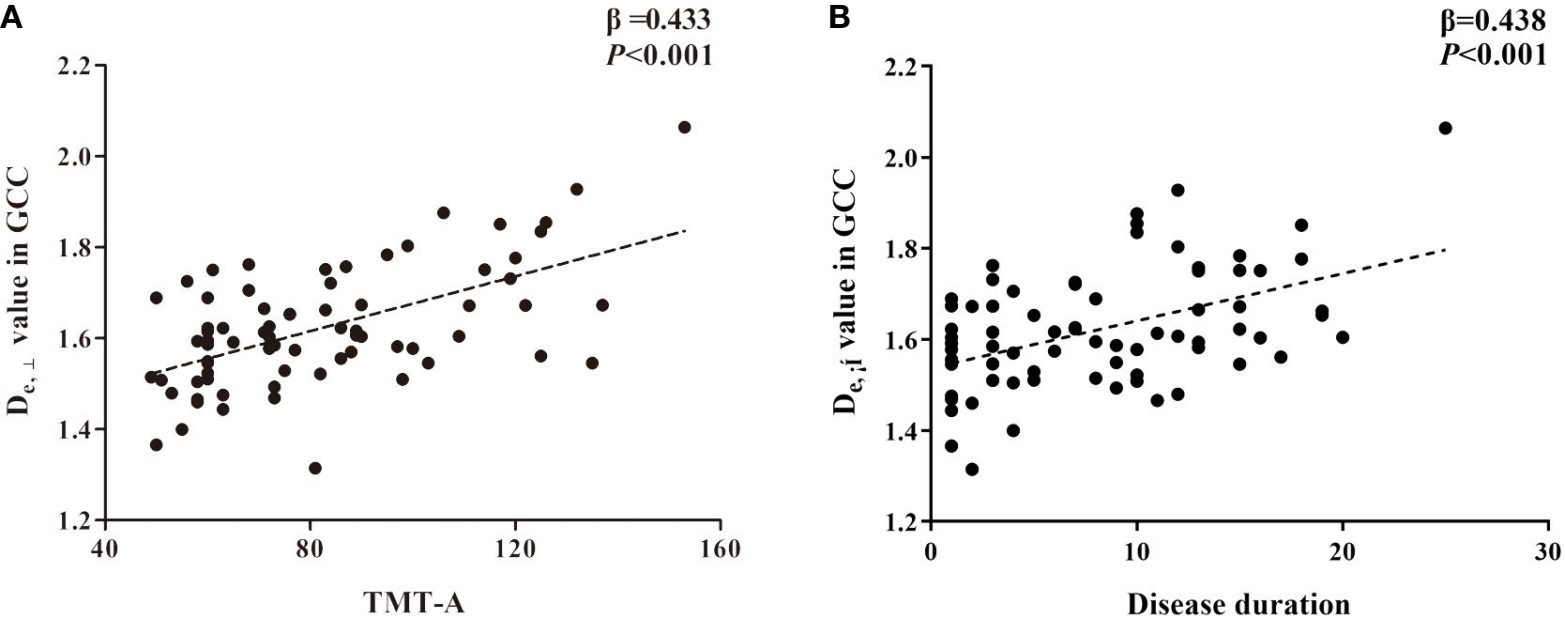
The significant correlations between D_e,⊥_ metrics in genu of corpus callosum (GCC) and cognitive **(A)** and clinical data **(B)** in T2DM patients based on ROI-wise analysis.

Based on the WM fiber tracts showing significant intergroup differences, we explored the relationships between diffusion metrics (FA and D_e,⊥_) of these tracts and the neuropsychological scores, disease duration, and HbA1c level in T2DM patients. The results showed that higher D_e,⊥_ values in the genu of the CC (GCC) were significantly correlated with worse performance in the TMT-A (β = 0.433, *P* < 0.001) and longer disease duration (β = 0.438, *P* < 0.001), as shown in **Figure 4**. Subsequently, we conducted additional analysis employing bootstrap methodology to examine the mediating influence of disease duration on the relationship between D_e,⊥_ values in the GCC and TMT-A. The findings indicated that the indirect impact of disease duration was not statistically significant (β = 0.041, 95%[CI] = [-.171, 0.253]). The comprehensive statistical outcomes are presented in **Table 4**. D_e,⊥_ in the resultant ROIs was not significantly correlated with the HbA1c level (R < 0.4 or *P* > 0.05). FA was not significantly correlated with any the neuropsychological scores, disease duration, or HbA1c level (R < 0.4 or *P* > 0.05).

**Table 4:** The mediating role of disease duration between D_e,⊥_ values in the GCC and TMT-A in T2DM patients.

Effect	Model 1			Mdoel 2			Mdoel 3		
	β	t	95%[CI]	β	t	95%[CI]	β	t	95%[CI]
D_e,⊥_ in GCC	0.433	3.978^***^	[0.216, 0.651]	0.438	3.557^***^	[0.192, 0.684]	0.415	3.488^***^	[0.178, 0.653]
Disease duration							0.041	0.387	[-.171, 0.253]
R^2^	0.391			0.220			0.392		
F	22.454^***^			9.896^***^			14.837^***^		

Model 1 represents the effect of D_e,⊥_ in GCC on TMT-A; Model 2 represents the effect of D_e,⊥_ in GCC on disease duration; Model 3 represents the effect of D_e,⊥_ in GCC and disease duration on TMT-A. ^***^P<0.001.

## 4 Discussion

The present study is the first to investigate WM microstructural changes in T2DM patients by using WMTI models based on DKI. This study demonstrated decreased FA and AWF and increased MD, D_e,//_, and D_e,⊥_ in widespread WM regions in T2DM patients. Additionally, our findings suggest that among all diffusional metrics, D_e,⊥_ is the most sensitive metric for detecting WM disruptions. In particular, the T2DM and HC groups showed only D_e,⊥_ differences in some crossing fibers, including the pontine crossing tract, bilateral PTR, right SLF, and bilateral UF. Additionally, D_e,⊥_ showed significant correlations with disease duration and cognitive performance, potentially providing valuable imaging evidence for predicting cognitive impairment.

Chronic brain damage in patients with T2DM has been a topic of concern, and understanding the pathogenesis of WM impairment in patients with T2DM has become increasingly important, since WM is believed play a prominent role in T2DM-induced cognitive impairment. Previous DTI studies employing TBSS, tractography, and voxel-based methods have consistently shown a reduction in FA and an elevation in MD within the WM of individuals with T2DM (7, 24). DTI is an effective modality for detecting the directional diffusion patterns of water molecules within tissues. FA and MD are two diffusion metrics commonly employed to quantify the directional preference of water molecules and average displacement within the WM (25), and have been widely recognized as significant composite metrics in the assessment of WM microstructural abnormalities. The observed decrease in FA and increase in MD may indicate compromised microstructural integrity of the WM (26, 27), which can be potentially attributed to various pathological processes such as ischemia, demyelination, axonal damage, inflammation, and edema (28). However, the ability of DTI models to accurately differentiate between these distinct pathological changes is limited in terms of specificity. To elaborate further, empirical diffusion measures solely offer indirect assessments of microstructure, thereby introducing uncertainty regarding their physical interpretation in relation to microscopic tissue parameters.

The WMTI model, based on DKI, provides more accurate and specific estimates of the microstructural brain environment by characterizing the intra-axonal and extra-axonal spaces. This allows for a comprehensive understanding of white matter changes from a biophysical perspective. In the model, the intra-axonal space represents impermeable myelinated axons, described by D_axon_, while the extra-axonal space represents the permeable medium of glial cells and myelin sheath, described by D_e,//_and D_e,⊥_. In addition, AWF, which characterizes the water fraction inside axons compared to total water fraction, maps axonal packing density and has been validated by electron microscopy images in mice (29). The utilization of WMTI metrics has proven advantageous in investigating WM alterations and potential pathogenesis in various clinical studies involving Alzheimer’s disease (14), multiple sclerosis (15), normal aging (16), and mild traumatic brain injury (17). In the present study, alongside the observed reductions in FA and increases in MD, significantly decreased AWF and increased D_e,//_and D_e,⊥_ were also identified in widespread WM regions of patients with T2DM. In terms of the interpretation of AWF, the decreased AWF could potentially be attributed to a reduction in intra-axonal water or an increase in the overall water content. Given that D_axon_ did not exhibit a decrease in this study, it is highly likely that the widespread decrease in AWF is primarily attributable to heightened diffusivity originating from the extra-axonal space. Moreover, it can be postulated that the elevated D_e,⊥_ observed in T2DM patients aligns with demyelination, while the increased D_e,//_signifies augmented permeability within the glial cellular environment, induced by hyperglycemia or inflammation. In addition, increased D_axon_ values were also found in some discrete WM regions, which may simultaneously reflect axonal injury in limited regions.

The aforementioned diffusion findings align with the histological characteristics observed through light and electron microscopy studies (30, 31), which indicated degenerative alterations in glial cells, disruption of the myelin sheath, fragmentation of neurofilaments, and abnormalities in oligodendrocytes in rats with T2DM (31, 32). These WM microstructural abnormalities can be explained by several possible mechanisms. Hyperglycemia is known to cause widespread impairment of microvascular function, including the brain’s microvasculature (33). This impairment may lead to inflammatory responses and reduced blood flow, ultimately resulting in chronic cerebral ischemia (34). Moreover, the elevated oxidative stress resulting from hyperglycemia plays a significant role in the development of endothelial dysfunction, facilitating the pro-inflammatory process (35). These pathogenic factors and pathways are highly likely to contribute to the dysregulation of cerebral blood supply. Notably, the WM is particularly susceptible to inadequate blood supply (36), and disruption of WM microstructural integrity (28) can be predominantly attributed to a combination of hyperglycemia-induced inflammation, hypoperfusion, oxidative stress, and endothelial dysfunction (36). We speculate that the initial pathologic change is mainly characterized by demyelination, and thus seemingly increased the D_e,//_, D_e,⊥_ and AWF across widespread WM regions. As neurodegeneration progresses, the loss of axons occurs secondary to demyelination, leading to the increased D_axon_ in limited WM regions.

This study showed a large degree of overlap in group differences between WMTI and DTI metrics. Consequently, this outcome serves as evidence of the dependability of the employed metrics in the identification of T2DM-associated microstructural abnormalities. Nevertheless, the DTI model assumes of a Gaussian distribution of water-diffusion processes with a mono-exponential signal decay. This assumption poses challenges in characterizing complex fibers and consequently limits the amount of information that can be obtained. In contrast, DKI allows for the estimation of the non-Gaussian distribution of water molecules and enables calculation of kurtosis parameters such as mean kurtosis, radial kurtosis, and axial kurtosis, which can provide insights into the structural complexity. For patients with T2DM, two studies have investigated brain microstructural integrity using DKI and have confirmed its greater sensitivity than DTI in detecting changes in brain microstructure (37, 38). Since WMTI metrics are derived from DKI, we hypothesized that they could offer supplementary insights into WM microstructural abnormalities. As expected, among the WMTI and DTI metrics, D_e,⊥_ detected more WM region differences in group comparisons, thereby exhibiting higher sensitivity and specificity for identification of subtle brain changes. Additionally, consistent with previous studies utilizing DKI, WMTI metrics also demonstrated remarkable capacity for imaging complex crossing fibers, such as the pontine crossing tract, PTR, SLF, and UF. While DKI metrics are sensitive to microstructural features, they lack specificity. Conversely, the D_e,⊥_ metric derived from the WMTI model provides a specific measurement of the extra-axonal compartments. Neurite orientation dispersion and density imaging (NODDI) is another multicompartment model utilized to distinguish the signal originating from extra- and intra-axonal compartments. A recent investigation employed NODDI to examine microstructural modifications in individuals with T2DM, and revealed a diminished intracellular volume fraction (Vic) in T2DM patients experiencing cognitive decline (39). A reduced Vic suggests a decrease in axon density, potentially indicating degeneration of axons and myelin. This is also consistent with our speculation regarding pathological changes. However, this study did not compare the sensitivity and specificity of the WMTI and NODDI metrics in detecting and monitoring microstructural alterations, since it would prolong the scan time and increase the burden for T2DM patients.

Furthermore, this study demonstrated that the D_e,⊥_ values in the GCC were correlated with cognitive function (TMT-A) scores and disease duration. To further analyze the role of disease duration as a possible mediator between the D_e,⊥_ values in the GCC and TMT-A scores, a mediational model was constructed. However, disease duration did not act as a mediator in this model, which contradicts previous beliefs that longer disease duration leads to more severe neurodegeneration and cognitive decline (5). This may be due to the relatively young age (55.64 ± 7.65 years) and short duration of illness (8.34 ± 5.93) in the current study. Diabetes-associated WM impairments have been observed to manifest prior to cognitive decline, even in individuals with prediabetes (40). Thus, in T2DM patients, impairment of WM microstructural integrity in various regions, particularly the frontal lobe, has been hypothesized to affect both structural and functional connectivity. This disruption may consequently lead to network disturbances and a subsequent reduction in neuronal signal transmission, resulting in slower information processing speed and cognitive decline. While T2DM is associated with a broad spectrum of WM microstructural disruptions, only a subset of these abnormalities appears to be linked to cognitive impairments, indicating the existence of important targets of cognitive impairment in the brain. The GCC, which serves as a principal commissural WM bundle connecting the left and right prefrontal lobes, is closely associated with cognitive function and plays a crucial role in the maintenance of cognitive processes such as attention and execution (41). Additionally, a previous study conducted on patients with prediabetes also indicated that the CC is particularly susceptible to hyperglycemia and may be one of the regions most severely affected by diabetes-related damage (40). Therefore, based on our findings, we propose that the observed increase in D_e,⊥_ within the GCC could potentially serve as an early imaging marker for assessing disease progression and monitoring cognitive function.

The findings in this study also have implications for the clinical management and risk prediction of cognitive decline in patients with T2DM. It has been widely accepted that T2DM was a risk factor associated with mild cognitive impairment and dementia. Cognitive decline starts slowly but can accelerate quickly. Therefore, tracking cognitive performance and changes of WMTI metrics over time and identifying a cut-off value for MCI could help clinicians identify patients with high risk of cognitive impairment early. These patients will receive personalized care, including strict glycemic control and management of cardiovascular risk factors. In addition, they can also benefit from early treatment with medications and cognitive behavioral therapy to prevent or delay cognitive decline.

This study had several limitations. First, this was a single-center study based on a Chinese population and only recruited inpatients with T2DM. Our results require validation in a larger and more diverse population. Second, this was a cross-sectional study, and the follow-up phase is still ongoing. Thus, longitudinal studies have not yet been performed due to the limited number of currently enrolled cases. Longitudinal studies evaluating the effectiveness of WMTI metrics would be useful in this regard. Additionally, patients with T2DM received medications, and we overlooked the effects of these medications on the brain. A partial reflection of medication effects cannot be ruled out in our results. Finally, we only focused on the WM impairments in patients with T2DM. In fact, WMTI metrics based on DKI should also have great potential for mapping changes in gray matter, which is worth further study in the future.

## 5 Conclusion

This study provides evidence that WMTI metrics exhibit greater sensitivity than DTI metrics in the detection of T2DM-related white matter microstructural abnormalities. The observed changes in WMTI metrics in T2DM indicate varying degrees of degeneration in the myelin sheath and axon. The WMTI model, as a noninvasive and sensitive technique, allows for early assessment of disruptions in white matter integrity among T2DM patients, potentially offering valuable insights into the underlying mechanisms of T2DM-related brain injury. Additionally, the correlation between D_e,⊥_ values in the genu of the corpus callosum and cognitive function and disease severity implies their potential role as imaging markers for monitoring disease progression and cognitive function in patients with T2DM.

## Data availability statement

The original contributions presented in the study are included in the article/supplementary material. Further inquiries can be directed to the corresponding author/s.

## Ethics statement

The studies involving humans were approved by The Human Ethics Committee of Shaanxi Provincial Peoples Hospital. The studies were conducted in accordance with the local legislation and institutional requirements. The participants provided their written informed consent to participate in this study.

## Author contributions

JG: Data curation, Formal analysis, Funding acquisition, Methodology, Writing – original draft, Writing – review & editing. PP: Formal analysis, Methodology, Data curation, Writing – review & editing. JL: Formal analysis, Software, Writing – review & editing, Validation. MT: Data curation, Formal analysis, Writing – review & editing, Validation. XY: Validation, Writing – review & editing, Data curation. XZ: Software, Validation, Visualization, Writing – review & editing, Data curation. MW: Formal analysis, Software, Validation, Writing – review & editing. KA: Methodology, Software, Writing – review & editing. XL: Resources, Supervision, Writing – review & editing. XiaonlingZ: Funding acquisition, Resources, Supervision, Writing – review & editing. DZ: Conceptualization, Funding acquisition, Investigation, Project administration, Resources, Supervision, Writing – review & editing.
